# Decreasing parental task specialization promotes conditional cooperation

**DOI:** 10.1038/s41598-017-06667-1

**Published:** 2017-07-26

**Authors:** Arne Iserbyt, Nolwenn Fresneau, Tiffanie Kortenhoff, Marcel Eens, Wendt Müller

**Affiliations:** Department of Biology, Behavioural Ecology and Ecophysiology Group, University of Antwerp, Universiteitsplein 1, B-2610 Wilrijk, Belgium

## Abstract

How much to invest in parental care and by who remain puzzling questions fomented by a sexual conflict between parents. Negotiation that facilitates coordinated parental behaviour may be key to ease this costly conflict. However, understanding cooperation requires that the temporal and sex-specific variation in parental care, as well as its multivariate nature is considered. Using a biparental bird species and repeated sampling of behavioural activities throughout a major part of reproduction, we show a clear division of tasks between males and females in provisioning, brooding and foraging. Such behavioural specializations fade with increasing nestling age, which stimulates the degree of alternated feeding visits, as a recently promoted form of conditional cooperation. However, such cooperation is thought to benefit offspring development, which is not supported by our data. Thus, from a proximate point of view, conditional cooperation via alternation critically depends on the division of parental tasks, while the ultimate benefits have yet to be shown.

## Introduction

Biparental care is relatively rare but appears taxonomically widespread across a number of invertebrate (e.g. some species of isopods, wood roaches, wasps and beetles) and vertebrate species (e.g. 90% of all birds, 23% of fish and 5% of mammals)^1, 2^. In such species, both parents provide resources that enhance offspring growth and survival, despite the fact that parents are typically unrelated. Therefore, each parent would profit when its mate invests more into their joint activity, as they only pay the costs of their own contribution^3^. Fundamental to this sexual conflict over parental investment is an allocation trade-off faced by each individual parent about the investment of time, energy and resources in current or future offspring. How much parental investment and provided by who, remain two puzzling questions since Trivers^4^ forged the foundation of this theory.

Conflict resolution may require high levels of behavioural flexibility in which each parent adjusts its own contribution in response to its partner’s contribution, cf. ‘negotiation’^5–7^. A constant monitoring of each other’s nest visits appears fundamental to fine-tune investment within pairs. This view is empirically supported in a number of bird taxa^8–12^, and recently inspired the development of an alternative theoretical model for conflict resolution^9^. More specifically, individual investment is enhanced when the partner was the last to visit and reduced when the individual itself was the last visitor, resulting in matched visit rates. Such flexibility in the rate and coordination of nest visits facilitates a rule of alternation in which parents take turns to feed their offspring. Alternated nest visits have been interpreted as a form of direct reciprocity^13^ or conditional cooperation^9, 11^, that may resolve the conflict over parental investment. In short, this strategy can be defined as ‘help someone who has helped you before’^13^. Maintaining high levels of alternation should thus ultimately be rewarded through an improvement of offspring development and physiological condition. However, the adaptive significance of this behavioural rule still needs to be confirmed by relating estimates for cooperation with parental and/or offspring fitness estimates^10, 12^.

Furthermore, whether parents are able to achieve equality in their investment via alternation is likely complicated by a number of important, but largely overlooked aspects. First, theoretical studies often modeled care as a univariate quantity, assuming equal and interchangeable units of male and female care^14, 15^. Empirical studies also tend to estimate parental investment by quantifying a very restricted number of care traits, typically offspring provisioning or incubation^1^. However, reproduction requires a large variety of direct (e.g. feeding or brooding) and indirect (e.g. nest defense or maintenance) behavioural tasks^16, 17^. Second, although parental care is a multidimensional trait, certain parental tasks can be sex-specific^18^. This raises the question how parents judge each other’s investment and how they coordinate overlapping activities. Third, offspring demand varies over the course of development, with direct consequences for parental tasks. In passerine birds for example, brooding behaviour is exclusively performed by females, but is gradually decreased with nestling age due to enhanced endothermic capacities of the nestlings^19, 20^. Temporal shifts in tasks may thus occur, which likely affect the way how male and female parents coordinate their activities and hence their level of cooperation. Despite this, such anticipated temporal variation in care remains conspicuously absent in theoretical studies^7^ and empirical studies that mostly focus on parental care and cooperation during a single snapshot in time^17, 21, 22^.

The above highlights that parental care is often (1) multidimensional, (2) sex-specific and (3) temporally variable, which may hamper any form of cooperation that relies on negotiation. These aspects have largely been overlooked thus far and demand detailed study to improve our current fragmented understanding of parental cooperation. We hypothesize that both sexual task specialization and the expected temporal variation in parental care limit the coordination of a shared task like food provisioning. We tested this hypothesis in a biparental bird species and quantified not only parental provisioning, but also a range of different behavioural activities during reproduction, their temporal variation, and sex-specificity. We then analyzed how this affects the coordination of a common task, here food provisioning. However, there is currently no good way to evaluate how well parents share and coordinate the overall work load of parental care, and hence the effects for sexual conflict remain unknown. We therefore related multiple aspects of parental investment and coordination with estimates of reproductive success. Such estimates include offspring development and physiological condition and can be interpreted as the result of negotiated overall work load between parents. One of the main expectations is that, if parents coordinate their rate of provisioning via alternation, the overall degree^10^ and temporal increase^7^ of alternation should ultimately enhance reproductive success.

## Methods

### Study species and animal keeping

We used a total of 92 adult Fife Fancy canaries (*Serinus canaria*) from our own outbred population. All individuals were one or two year old during the experiment. Five weeks prior to the breeding period, males and females were housed separately in two large indoor aviaries. All birds experienced a long light regime during this period (14:10, L:D) and had access to seeds and water *ad libitum* (see comment discussion). Egg food was provided twice a week. Next, males were housed in individual cages (50 × 64 × 40 cm, GEHU cages, The Netherlands) for three days. Thereafter, each male was paired by allocating an unrelated female to the cage, and nesting materials were provided. Progress on nest building, egg laying (mean ± SE clutch size = 3.97 ± 0.17 eggs) and incubation (12.5 ± 0.16 days) was monitored daily^23^. Hatching was synchronized within broods by keeping the first two eggs at room temperature (20 °C) and returning them after the third egg was laid^24^. This reduced age and size asymmetry among nestlings, which equalized competitive abilities among siblings and moderated brood reduction. Enriched egg food was provided on a daily basis after hatching (day 0). Nestlings were individually marked with a unique within-nest colour on their back, using non-toxic pens (Artline®70 N), which was reapplied when necessary. Metal leg rings with a unique code were attached when a minimum of 5 g was reached. All nestlings were weighed daily from day 0 until day 14, which corresponds with the linear phase of nestling growth^25^. Nestling independence is reached after 25 days. On this day, fledgling body mass was measured with a digital balance (Kern EMB200-2; accuracy 0.01 g) and tarsus length with a digital caliper (Digimatic, Mitutoyo; accuracy 0.01 mm). Both metrics can be used to calculate fledgling condition in various ways, for example as the scaled mass index^26^ or as the regression residuals of body mass and tarsus length^22, 27^. Both condition estimates gave similar statistical outcomes and we here present the latter estimate. Furthermore, we calculated nestling survivorship per nest as the proportion of successfully fledged nestlings relative to the original number of hatchlings on day 1. The above procedure was followed for 21 pairs in spring 2015 and 25 pairs in spring 2016. Slight differences in approach between both years include assessment of additional behavioural categories and a higher number of repeated measurements in 2016 (see further), as well as a standardized parental work load (i.e. 4 nestlings per brood) in 2016. Specifically for the latter, partial cross-fostering at an early stage (within 24 h post-hatching) occurred in most (20/25) nests, so that each nest contained four nestlings. In these cases, at least one nestling was relocated with a maximal age difference of 12 h between nestlings, to equalize competitive abilities within nests. Furthermore, all nestlings hatched from eggs with a different egg order, to equalize maternal effects across nests^23^. Cross-fostering is known to have limited effects on parental behaviours and offspring survival in our population^24^. Year and brood size were always included in our statistical modelling (see further).

### Behavioural measurements

**Figure 1:**
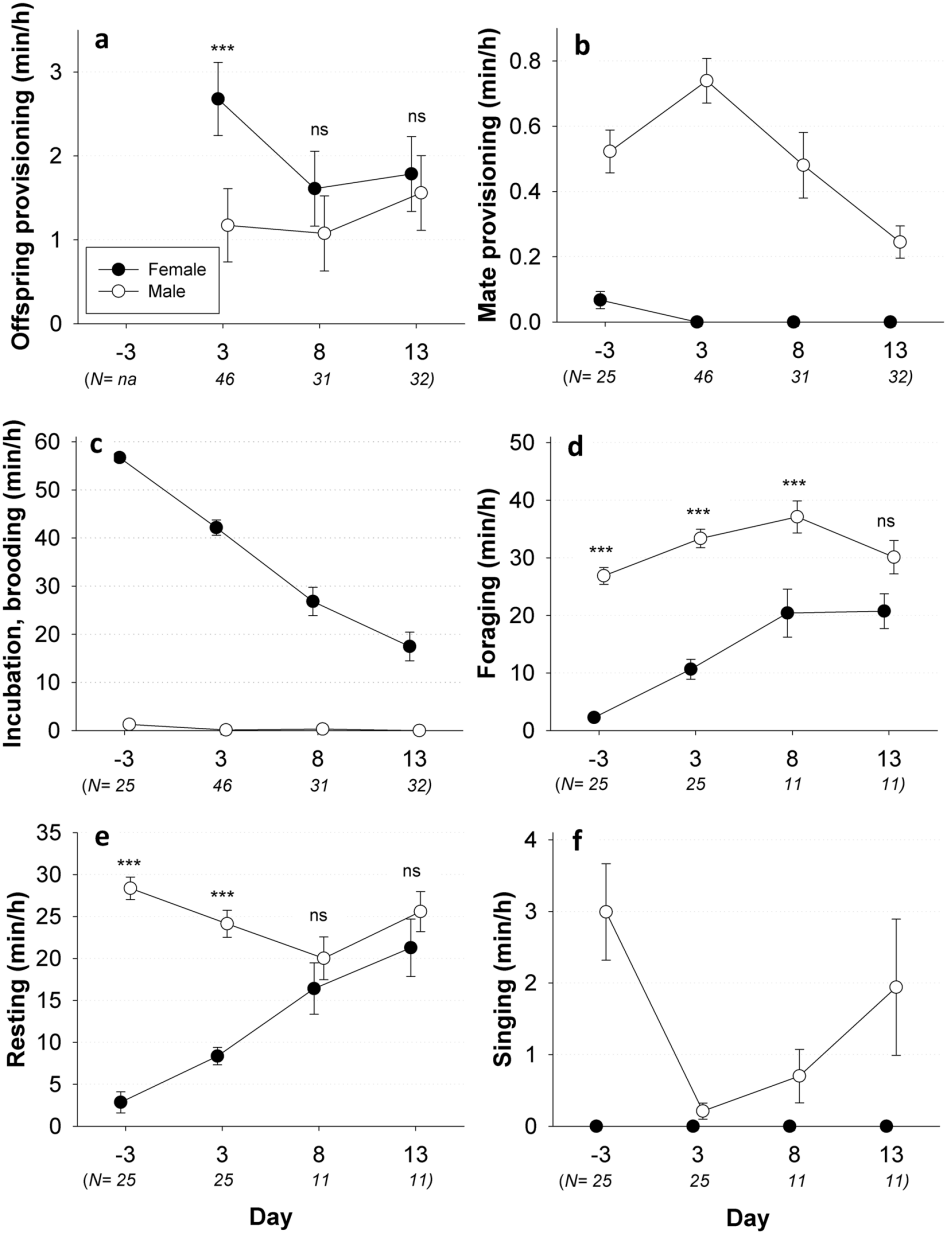
Temporal and sex-specific variation in behavioural activities. Mean (±SE) time per hour allocated to six different behavioural traits by males (white dots) and females (black dots). These observations were made on four distinct days, with ‘−3’ representing three days prior to the expected hatching date. Post-hatching days 3, 8 and 13 represent the average nestling age. Sex-differences per day were evaluated via Tukey corrected post-hoc tests, with ***indicating P < 0.0001. This was not done for mate provisioning (**b**), incubation or brooding (**c**) and singing (**f**) behaviour, because these behaviours are nearly exclusively associated to one sex. Number of observed nests (N) used to quantify each trait is indicated in brackets below the x-axes.

Behavioural activities of the parents were assessed from 1.5 h (89.4 ± 0.81 min, range [50.5–92.5 min], N = 134) video recordings, which provides a good estimate given the high repeatability for behavioural traits in such a setup (R ≥ 0.57)^28, 29^. Recordings were always made between 10am and 2 pm to reduce any possible bias in diurnal activity. To capture the temporal dynamics of these behavioural activities, video recordings were repeated on four specific days. Pairs were video monitored 3 days prior to the expected hatch date (only in 2016, further indicated as day −3) and when nestlings were 3, 8 and 13 days of age. This covers the major part of the reproductive event. Prior to each video recording, the foraging motivation was standardized across nests by refilling the seed containers, and by additionally providing fresh green and egg food. Videos were analyzed in detail (to the nearest 0.1 s) using The Observer XT10.5 (Noldus, The Netherlands). The entire observation was partitioned into six behavioural categories for both parents separately. Specifically, these six behavioural traits were: (1) provisioning of predigested food, directly to the offspring, (2) provisioning of predigested food to the mate, (3) incubation (day −3) or brooding (observations post-hatching) when direct body contact was made with respectively the eggs or nestlings, (4) foraging as eating seeds, green food, egg food or drinking water, (5) resting as being perched for at least 1 s or preening feathers, and (6) singing with a minimum song bout length of 1 s. The behaviours 1, 2 and 3 are considered as forms of parental care that directly benefit the offspring. The other behaviours indicate a combination of indirect care (behaviour 4), self-maintenance (4 and 5), territory defense and mate attraction (6)^30^. Time allocated to these behaviours were for each individual corrected for the variable length of the video recording, and is presented as minutes per hour throughout. Foraging, resting and singing behaviour could only monitored in 2016, because the camera was then positioned on the entire cage and not only on the nest cup as in 2015. The number of nests monitored in 2016 on day −3 and day 3, was higher compared to days 8 and 13, as some nests were allocated to a different experiment that started at day 7. The exact number of observations per behaviour and day are detailed in Fig. 1.

Alternation was calculated based on the number and order of parental nest visits, hence direct male and female offspring provisioning bouts. The proportion of alternated visits (A) was calculated as in Bebbington & Hatchwell^10^ using the formula:

1 $$A=\frac{F}{{T}_{m}+{T}_{f}-1}$$

in which F is the number of alternated nest visits, independent of sex and T is the total number of nest visits for males (T_m_) and females (T_f_). To test whether pairs alternated more than expected by chance, we calculated the expected alternation (A_exp_) score for each individual pair and day, given the observed number of male and female nest visits. This expected alternation score is calculated as:

2 $${A}_{exp}=\frac{{A}_{max}+{A}_{min}}{2}$$

A_max_ is the theoretical maximum proportion of alternated nest visits, which is restricted by the number of visits by the least visiting parent (T_min_) and is calculated as:

3 $${A}_{max}=\frac{2{T}_{min}}{{T}_{m}+{T}_{f}\,-\,1}$$

In cases where T_m_ is equal to T_f_, this maximal proportion is set to 1. Finally, A_min_ is the theoretical minimum proportion of alternated nest visits which is calculated as:

4 $${A}_{min}=\frac{1}{{T}_{m}+{T}_{f}\,-\,1}$$

A_min_ resembles a hypothetical situation in which one parent successively visits the nest, followed by the other parent that successively visits the nest. Our data also allows to calculate the proportion of synchronized nest visits, as a second proxy for parental cooperation. All the details on this proxy can be found in Supplementary material 1.

### Offspring physiology

At independence (day 25), a 50 µl blood sample was collected from the alar wing vein for molecular sex determination. CHD-genes were amplified by PCR using the protocol detailed in Griffiths *et al*.^31^.

The nestling blood sample further allowed to quantify plasma nitric oxide (NO) levels. This small molecule yields a multitude of physiological functions, and has been suggested to signal individual status of immune functioning and oxidative stress^32, 33^. Hence, assessing NO levels can provide useful information on nestling physiological condition and health state. To quantify the concentrations of NO (µmol/L), a spectrophotometric assay was used which is fully detailed in Sild & Hõrak^33^. All samples were analyzed in duplicate and were quantified using a standard curve, included within each 96-well plate.

### Compliance with ethical standards and data availability

The approved experiment was carried out in accordance with the guidelines of the Ethical Committee of the University of Antwerp, Belgium (ID: 2012-51 and 2015-64). The dataset supporting this article was uploaded to the Dryad digital repository doi:10.5061/dryad.gb1vp.

### Statistical analyses

In a first set of analyses, we ran six separate General Linear Mixed Models (LMMs, lme4 package)^34^ to test whether variation in time allocated towards a given behaviour was explained by parental sex differences, nestling stage, their interaction, and the number of eggs or nestlings. Sex was excluded from the analysis for mate provisioning, incubation/brooding and singing behaviour, because these traits are nearly exclusively associated to one sex. We included three random intercepts in these models. Bird identity (BirdID) nested within nest identity (NestID) was included to avoid pseudo-replication of repeated measurements per individual. NestID was included because male and female behaviours are not independent as they share the same nest. Year was also included, to account for potential differences in breeding conditions. A similar model was run for alternation, using the above explanatory variables except for sex as fixed effect and BirdID as random effect, since alternation is not a trait at the individual but at the pair level. To contrast the observed with the expected alternation scores, an additional LMM was performed including two categorical fixed factors, nestling age and data type (categorical: observed or expected) along with their interaction. Sex-differences were assessed for each day with Tukey-corrected post-hoc tests (lsmeans package)^35^. Normality assumptions were always confirmed via Shapiro tests, except for male song behaviour. In this case we performed a zero-inflated generalized linear mixed model (glmmTMB package)^36^, including the same dependent and independent variables as described above.

In a second set of analyses, three separate LMMs were performed to test whether both overall^10^ and temporally varying^7^ levels of parental care behaviour and cooperation were predictive for our offspring fitness estimates. These three models contained either individual nestling growth rate, fledgling condition or fledgling NO level as dependent variables. Explanatory variables included nestling sex, brood size at day 13 and two main categories of behavioural variables that could directly affect offspring development. The first category comprised overall (mean of day 3, 8 and 13) levels of direct and indirect provisioning, brooding and alternation scores. The second category indicated the individual change in each of these behaviours. This was done for each individual or pair by calculating their slope when each specific behaviour was regressed against day as continuous variable. Year and NestID were added as random effects. Finally, a Generalized Linear Mixed Model with binomial distribution and a logit link function (glmer function)^34^ was used to relate the proportion of successfully fledged nestlings with the overall and temporally varying predictor variables detailed above, plus the original number of nestlings on day 3. Our approach may raise potential multicollinearity problems among the predictor variables. These were never severe, except when averaged female brooding was included (variance inflation factor, VIF = 5.78). Excluding this variable resulted in maximal VIF values that never exceeded 1.99. The effect of overall level of female brooding on the offspring fitness estimates were then tested separately in parallel models.

All analyses were performed in R (version 3.2.2)^37^. As described and advised by Zuur *et al*.^38^, All models were reduced using backward stepwise elimination of the least significant (highest P value) fixed effects, until only significant (P ≤ 0.05) variables remained. Alternative model selection procedures based on AIC values were not used, given the large number of alternative models (inflated type-I errors by multiple testing) and the difficulty to cope with missing values^39^.

## Results

### Variation in behavioural activities and coordination

Males and females showed striking differences in all six assessed behavioural traits (see Fig. 1; Table 1), three of which were nearly exclusively expressed in one sex. Incubation and brooding on the one hand was typically performed by females (Fig. 1c). Only short (2.11 ± 0.34 min/h) bouts of incubation were observed in 15 out of 25 males, while male brooding occurred in three anecdotal observations. Mate provisioning (Fig. 1b) and singing (Fig. 1f) on the other hand were nearly exclusively performed by males. The other three assessed behavioural traits, offspring provisioning, foraging and resting were commonly observed by both sexes, but the former behaviour was predominated by females and the latter two by males (Fig. 1a,d,e; Table 1).

**Table 1:** Statistical outcome of the six mixed models explaining temporal and sexual variation in behavioural activities.

	Effect	DF	F/z	P
(a) *Offspring provisioning*				
	Brood size	1, 185.6	0.00	0.9848
	Sex	1, 76.7	21.8	**<0.0001**
	Day	2, 151.1	8.81	**0.0002**
	Sex*Day	2, 130.9	12.9	**<0.0001**
(b) *Mate provisioning - males*				
	Clutch/Brood size	1, 105.2	5.01	**0.0274**
	Day	3, 88.3	6.35	**0.0006**
(c) *Incubation*, *brooding - females*				
	Clutch/Brood size	1, 124.4	0.03	0.8531
	Day	3, 98.0	50.5	**<0.0001**
(d) *Foraging*				
	Clutch/Brood size	1, 111.3	0.56	0.4579
	Sex	1, 30.3	91.6	**<0.0001**
	Day	3, 101.9	22.9	**<0.0001**
	Sex*Day	3, 99.1	8.35	**0.0001**
(e) *Resting*				
	Clutch/Brood size	1, 125.4	8.38	**0.0045**
	Sex	1, 58.0	62.2	**<0.0001**
	Day	3, 108.7	2.00	0.1183
	Sex*Day	3, 103.4	18.5	**<0.0001**
(f) *Singing - males*				
	Clutch/Brood size	—	−1.11	0.2660
	Day	—	−4.01	**<0.0001**

These are full model outcomes, but stepwise removal of the non-significant clutch/brood size effect did not alter any of the significant temporal and sexual effects. Note that mate provisioning, incubation/brooding and singing are nearly exclusively associated to a particular sex, hence these analyses were restricted to one sex. Analyses (a–e) are general linear mixed models, while analysis (f) is a zero-inflated generalized linear mixed model.

In addition, significant temporal variation was observed in all six assessed behavioural traits (Table 1, Fig. 1). This temporal variation clearly differed between males and females, at least for the three behavioural traits that allowed sexual comparisons (see Table 1; sex*day interactions: all P < 0.0001). Particularly, the pronounced sex differences in offspring provisioning, foraging and resting that occurred during the early phases of reproduction gradually faded with increasing nestling age. Specifically, offspring provisioning was more than twice as high in females, relative to males on day 3 (z = 7.54; P < 0.0001; Fig. 1a), but this significant difference was absent on day 8 (z = 2.23; P = 0.22) and day 13 (z = 0.96; P = 0.93). Similar observations were found for foraging (significant male-female differences on day −3, 3 and 8: all P < 0.0001, but not on day 13: z = −2.71; P = 0.12; Fig. 1d) and resting (day −3 and 3: both P < 0.0001, day 8: z = −1.51; P = 0.80, day 13: z = −1.48; P = 0.82; Fig. 1e). Sex-specific behaviours, as male mate provisioning and female brooding substantially decreased from day 3 to day 13 and are hence consistent with the other observed decreasing differences in behavioural activities with increasing nestling age.

Egg or nestling number had a positive effect on male mate provisioning (estimate: 0.15 ± 0.07; P = 0.027) and a negative effect on resting behaviour (estimate: −3.68 ± 1.27; P = 0.004; see Table 1). The four other behavioural parameters were not significantly influenced by number of eggs or nestlings.

**Figure 2:**
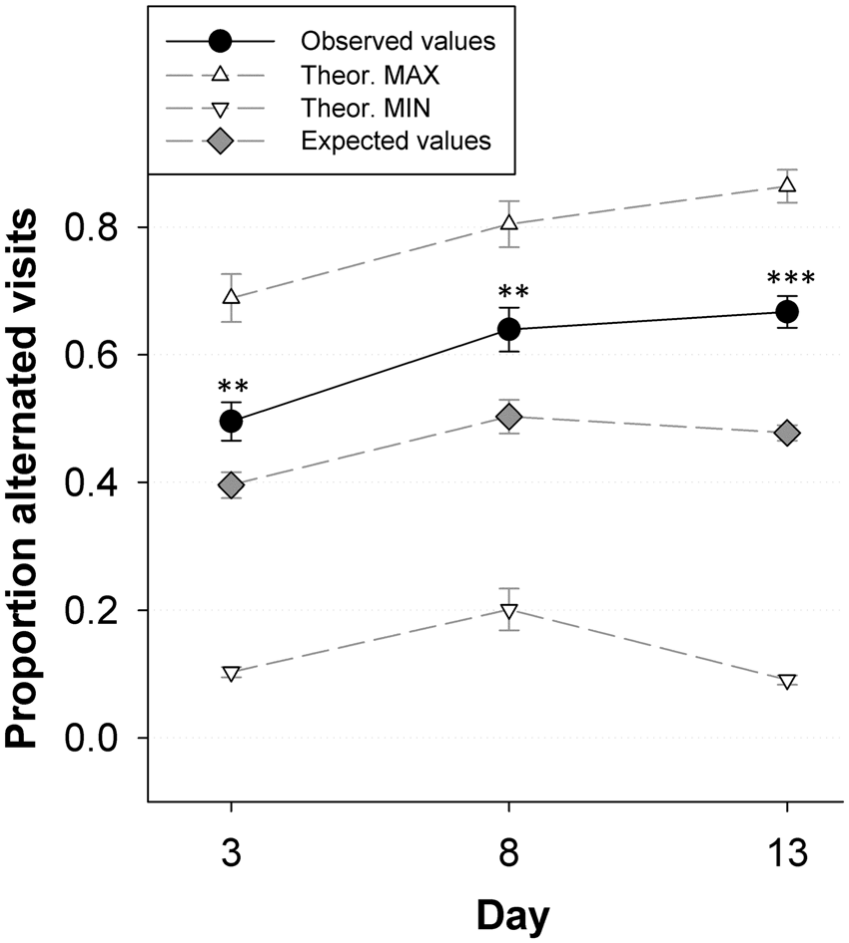
Temporal variation in nest visit alternation. Observed values are population averages (±SE) per day and are represented by the black dots and solid line. The mean between the theoretical maximal (white triangles pointing up) and minimal (white triangles pointing down) alternation value was calculated for each observation and is considered the expected proportion of alternated nest visits by chance (grey diamond symbols), taking into account the observed parental nest visits (details see methods). The observed proportion of alternated visits increased with nestling age and were consistently higher than the expected values. Daily Tukey-corrected post-hoc differences between empirical and expected samples are indicated as **(0.01 < P < 0.001) and ***(P < 0.001).

Significant temporal variation was observed in the degree of alternation, as turn taking clearly increased with nestling age (F_2,70.9_ = 11.1, P < 0.0001; see Fig. 2). Tukey corrected post-hoc tests revealed a significant increase in alternation from day 3 to day 8 (∆14.40%; z = −3.55, P = 0.001), while no difference was observed in alternation from day 8 until day 13 (∆2.76%; z = −0.61; P = 0.81). When compared against the expected alternation scores, observed values were consistently (Nestling Age*DataType: F_2,157.4_ = 1.95; P = 0.15; see methods) and significantly higher than the expected values (F_1,159.5_ = 50.50, P < 0.001). Specifically, the observed values were higher than the expected values when nestlings were 3 days (∆9.93%; z = 3.40; P = 0.009), 8 days (∆18.95%; z = 3.77; P = 0.002) and 13 days old (∆24.80%; z = 5.41; P < 0.001; see Fig. 2). The number of nestlings did not have an effect on parental alternation (F_1,66.6_ = 0.079, P = 0.80).

We then tested whether total nest visit rates (number of direct provisioning bouts) was related to alternation, sensu^9, 10, 40^. Firstly, we found that the temporal variation in nest visit frequency differed between sexes (Sex*Day interaction: F_2,130.0_ = 26.1; P < 0.0001). Females, relative to males, visited the nest more often on day 3 (5.1 ± 0.3 vs 2.9 ± 0.3 visits/h; z = 6.48; P < 0.0001), equally on day 8 (3.2 ± 0.3 vs 2.9 ± 0.4 visits/h; z = 0.76; P = 0.45) and less often on day 13 (3.9 ± 0.2 vs 5.1 ± 0.4 visits/h; z = −3.11; P = 0.002). The mean skew in visit rate within pairs as absolute values was 2.8 ± 0.3 (day 3), 1.6 ± 0.2 (day 8) and 1.8 ± 0.3 (day 13) visits/h. Secondly, alternation scores were not related to total visit rates, neither as an interaction with nestling age (F_2,92.9_ = 0.06; P = 0.95), nor as a main effect (F_1,93.0_ = 1.08; P = 0.30). Similar observations were found for total time spent provisioning (interaction with nestling age: F_2,90.5_ = 0.006; P = 0.99; main effect: F_1,86.9_ = 0.50; P = 0.48).

### Variation in offspring fitness estimates

**Figure 3:**
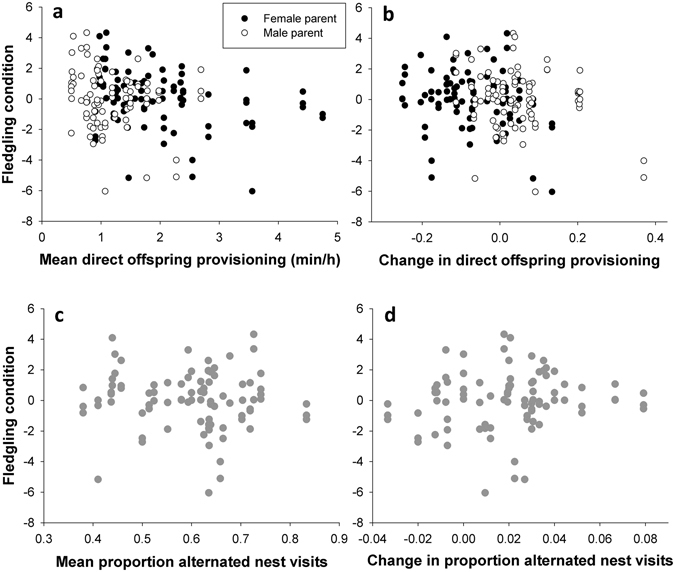
Fledgling condition as illustrative example for the absent relationships between offspring traits and estimates of parental care. Fledgling condition is here presented as the mass-by-tarsus length regression residuals, with positive values indicating heavy birds relative to their tarsus length and *vice versa*. Mean individual male (white dots) and female (black dots) provisioning (**a**) and pair (grey dots) alternation scores (**c**) are averaged values across three days (day 3, 8 and 13). Individual changes in both traits (**b**,**d**) are characterized as the slope of the behaviour-by-day regression. Fledgling condition was neither related to male mate feeding and female brooding and moreover, behavioural effects were consistently absent for the other offspring fitness parameters (all P > 0.05), except for NO levels (see results).

Offspring growth rate, fledgling condition and nestling survival were not significantly affected by any of the parental behaviours, neither as average values (direct male and female provisioning: all P ≥ 0.08; mate provisioning: all P ≥ 0.66; female brooding: all P ≥ 0.16 and alternation: all P ≥ 0.23), nor as changes from day 3 until day 13 (respectively, all P ≥ 0.16; ≥0.54; ≥0.08; ≥0.13; see Fig. 3). Similar observations were made for fledgling body mass. Most of the behavioural parameters did not influence NO levels, except for a negative effect of average male mate feeding (F_1,80.11_ = 6.35; P = 0.014) and an opposite stimulating effect by the change (mainly degree of increase) in male offspring feeding (F_1,80.28_ = 14.30; P = 0.0003). Brood size had no effect on any of these offspring measurements. Nestling sex differences were observed in growth rate (F_1,80.14_ = 4.16; P = 0.045), fledgling body mass (F_1,74.67_ = 9.02; P = 0.004), but not in fledgling condition (F_1,80.7_ = 0.32; P = 0.57). Male nestlings generally grew faster (0.84 ± 0.12 g/day) and had a higher body mass (17.55 ± 1.71 g) than females (respectively, 0.77 ± 0.13 g/day; 16.55 ± 1.72 g).

## Discussion

Using canaries as a biparental model species, we have shown that parental care comprises multiple tasks and that both sexes are specialized in performing such tasks. These task specializations appear to constrain the degree of alternated coordination of a common task, here food provisioning. This is also reflected by the fact that alternation during offspring provisioning increased when the degree of task specialization faded over the course of the reproductive period. However, this estimate for parental cooperation has at best a limited effect on offspring development, physiological condition and survival, despite our observations that alternation varies strongly among pairs and that parents generally alternate more than expected by chance. The implications of these observations are discussed.

### Dynamic multitasking parents

Sex roles in care are omnipresent in nature, with taxon-specific strategies ranging between strictly uniparental male and uniparental female care^2, 41–43^. Within biparental species, however, such sex roles or behavioural task specializations also occur^20, 44^, but are frequently ignored. Our detailed study confirms that parental care is multidimensional, with parental behaviours forming a continuum from common tasks to exclusively male or female tasks. Specifically, egg incubation and nestling brooding behaviour are (nearly) exclusively performed by females. Males, in contrast, engage more in mate provisioning, which probably also explains their higher foraging effort. They also rest more than females and exclusively perform song behaviour. The latter reflects, among other functions, investment in territorial defense^30^.

A likely explanation for these sex-specific behaviours is the difference in efficiency in performing each behavioural task^18^. For example, only canary females have a distinct brood patch that guarantees an efficient heat transfer during incubation and brooding^45^. This sexual plumage dimorphism, along with the associated sexual asymmetry in costs, may explain why females invest most of their time in incubation and brooding compared to males. However while incubating, females heavily rely on food provided by their mates. This division of tasks makes males and females mutually dependent on each other to successfully reproduce, which supports recent theoretical predictions^18^. The synergistic costs of performing multiple tasks, combined with a sexual asymmetry in these costs are expected to facilitate a self-reinforcing co-evolutionary process between the degree of task specialization and the stability of biparental care^3, 46^. Up to date, empirical studies testing such relationships remain conspicuously absent^47^, but our exploratory study may be an initial step forward.

We found remarkable temporal variation in all behaviours, with as general pattern that task specializations gradually faded with increasing nestling age. This pattern is probably driven by the gradual increase of the nestling thermoregulatory capacity, which progressively frees the females from brooding. Such temporal patterns in behaviour have to be taken into account, especially for the interpretation of studies that provide a single behavioural snapshot in time when estimating parental investment and the degree of cooperation^7, 20, 48^. Furthermore, the repeatability of our six parental behaviours ranged from low (mate provisioning: R = 0.07) to high (foraging: R = 0.58), but both estimates of parental coordination were low (alternation: R = 0.15; synchronization: R = 0.03; Supplementary material 2). This further strengthens the urge to include temporal dynamics of parental strategies in future studies, and to carefully consider the time windows between repeated measurements^49^.

### Implications for conditional cooperation in birds

One of the implications of the observed task specialization is that male and female visit rates are unequal during early reproduction, ultimately resulting in low alternation scores. Our observations therefore suggests that task specializations constrain the degree of coordination of common tasks, like food provisioning. The outcome of our study corresponds with observations in long-tailed tits, *Aegithalos caudatus*, where an asymmetry in male and female visit rate led to a decreased degree of alternation^10^.We observed a temporal increase in alternation scores, which were always significantly higher than expected by chance. Alternation thus appears to be a behavioural phenomenon that is usually present between caring parents^9, 10^, but this form of cooperation hinges on the condition that parental tasks are similar and shared. Whether and how investment in other, non-overlapping tasks is coordinated between parents remains unknown. To date, it is thus not yet clear whether alternation is a valid behavioural strategy to facilitate parental cooperation. We therefore encourage future studies to experimentally manipulate investment across tasks and to assess its impact on a suite of male and female parental tasks.

### Adaptive behavioural strategies?

Contrary to observations in great tits, *Parus major* ^9^ and long-tailed tits^10^, we found that nest visit rate and alternation were not related. Neither did we find a relationship between time spent offspring provisioning and alternation. This suggests that both behaviours in canaries can be interpreted as independent estimates for respectively, parental investment (i.e. quantity) and parental cooperation (i.e. coordination). Alternation in our case study is thus not facilitated by matched male and female visit rates, which questions whether this behavioural mechanism tempers the conflict between caring parents^9^. We believe that the contrasting findings may result from species-specific differences in the adequacy of information gathering about partner effort^23, 50^. The variation in caterpillar size delivered by great and long-tailed tit parents (single-load species) is probably smaller than the variation in amount of processed and transferred seeds by canary parents (multi-load species) during a single feeding event. Estimating partner investment via visit rates may be particularly difficult in such multi-load species, given the greater potential for cheating during food transfer. Hence, this makes the anticipated link particularly difficult between alternation, as a signal of investment towards the partner, and the true level of parental investment.

We found no effects of both overall and flexible levels of parental investment (direct and indirect provisioning and brooding) and parental coordination (alternation and synchronization, see supplementary material) on offspring development, survival and particularly limited effects for offspring physiological condition. Parental behaviours had thus at best a limited effect on offspring development in our study system. One could argue that offspring fitness estimates in captivity with optimal housing conditions do not reflect offspring fitness in nature. However, our previous work with captive canaries highlights particularly large variation in offspring development (up to 25% difference in average fledgling body mass) and survival across nests, despite the *at libitum* food conditions^23, 25, 51^. This most likely reflects large variation in parental quality and the ability to cope with the costs associated with foraging and provisioning, despite such favourable conditions. That said, very few studies assessed the adaptive significance of alternation so far. In line with our observations, cooperative breeding acorn woodpeckers, *Melanerpes formicivorus*, were found to significantly alternate their visits with other group members, but such coordination did not affect social status, group coherence or mating probabilities^11^. Neither did alternation predict male or female survival in long-tailed tits^10^, although a positive relationship was found between alternation and fledgling success. Studies that related fitness estimates with synchronization, another estimate for parental cooperation (see also Supplementary material 1), are also scarce and have rendered ambiguous outcomes. In line with our findings (Supplementary material 1), the degree of synchronization had no effects on offspring quality traits and fledgling success in long-tailed finches^12^ and long-tailed tits^10^, but had positive effects on fledgling success in wild zebra finches, *Taeniopygia guttata* ^52^. Taken together, the empirical support for the hypothesis that nest visit coordination is beneficial for the offspring is limited or at best equivocal. This questions whether alternation represents a form of conditional cooperation, as theory predicts higher levels of parental care and thus offspring benefits among pairs that alternate well^9^.

Finally, we suggest that future research should incorporate measures of parental fitness in addition to measures of offspring fitness. It may well be that the parents and not the offspring, bear the costs of conflict^18^. More particularly, by maintaining an alternation rule that is always significantly higher than expected, parents may suffer from reduced condition to constantly signal their efforts to each other, while offspring condition may remain unaffected. Future studies should also consider that each parent may not only adjust its contribution based on the amount of care provided by its partner^5, 53^, but also based on its own state^17^, its partner’s state^7^ or various (a)biotic environmental heterogeneities^8, 54^. Behavioural flexibility is thus key to comprehend the stability of family life and we here stimulate future research to address hypotheses in a reaction norm framework^54^.

## Electronic supplementary material

Supplementary Material
