# Supplementary material for: Decreasing parental task specialization promotes conditional cooperation
Source: Sci Rep. 2017 Jul 26;7:6565. doi: 10.1038/s41598-017-06667-1 (PMC5529357; doi:10.1038/s41598-017-06667-1)
Supplement: Supplementary file 1 — Supplementary Material [file 41598_2017_6667_MOESM1_ESM.pdf]

Supplementary material

Title: Decreasing parental task specialization promotes conditional cooperation

Authors: Arne Iserbyt[a]*, Nolwenn Fresneau[a], Tiffanie Kortenhoff[a], Marcel Eens[a] & Wendt Müller[a]

Affiliation: [a] Department of Biology, Behavioural Ecology and Ecophysiology Group, University of Antwerp, Universiteitsplein 1, B-2610 Wilrijk, Belgium.

* **Corresponding author**:

Email: arne.iserbyt@uantwerpen.be

Tel: +3232652347

Fax: +3232652271

Supplementary material 1 - Synchronization

Besides alternation, nest visit synchronization has been suggested as another form of parental cooperation[1–3]. Adaptive hypotheses for synchronization include improved exchange of information and signaling individual investment among care takers, equitable food distribution among offspring and reduced activity around the nest to minimize predator attention[2]. We here performed the same statistical analyses as described in the main document for alternation, but now assessing temporal variation in observed and expected levels of synchronization, as well as its impact on our four offspring fitness estimates.

Mathematical and statistical approach

Observed levels of synchronization (S) were calculated as twice the number of synchronous male and female visits (overlapping direct provisioning bouts, S_n) upon the total number of male and female nest visits:

$$S = \frac{2S_n}{T_m + T_f} \tag{1}$$

Similar as for alternation, an expected synchronization score could be calculated for each observation as the average between the theoretical maximum (S_{max}) and theoretical minimum (S_{min}) synchronization scores. This maximum depends on the number of visits by the least visiting parents (T_{min}), for which all visit bouts ideally show overlap with its mate and is calculated as:

$$S_{Max} = \frac{2T_{Min}}{T_m + T_f} \tag{2}$$

The theoretical minimum synchronization score is 0 in all cases, representing an observation period in which direct offspring provisioning never overlaps.

All the performed statistical models for synchronization are identical as the ones for alternation and are described in detail in the main document.

Results

Parental nest visit synchronization significantly decreased ($F_{2,73.4}$ = 4.46, P = 0.015) with increasing nestling age (see supplementary material Fig. 1). Specifically, synchronization tended to decrease from day 3 onwards (relative to day 8: Δ -8.90%, z = 2.19; P = 0.072 and day 13: Δ -11.72%, z = 2.77, P = 0.015) and remained rather constant from day 8 towards day 13 (Δ -2.82%, z = 0.50, P = 0.87). In contrast to the observed synchronization values, the expected values increased with nestling age (Nestling Age*DataType: $F_{2, 105.0}$ = 6.13; P = 0.003; Supplementary material Fig. 1). Specifically, the observed synchronous nest visits were similar to the expected values on day 3 (Δ -4.69%; z = -1.31; P = 0.77), but were much lower on day 8 (Δ -18.45%; z = -4.23; P = 0.0003) and day 13 (Δ -24.67%; z = -5.84; P < 0.0001). The number of nestlings had no effect on nest visit synchrony ($F_{1,64.8}$ = 0.057, P = 0.81). Synchronization scores were not related to total (male plus female) visit rates, not as an interaction with nestling age ($F_{1,94.04}$ = 1.42; P = 0.24) and neither as a main effect ($F_{1,87.7}$ = 0.39; P = 0.53).

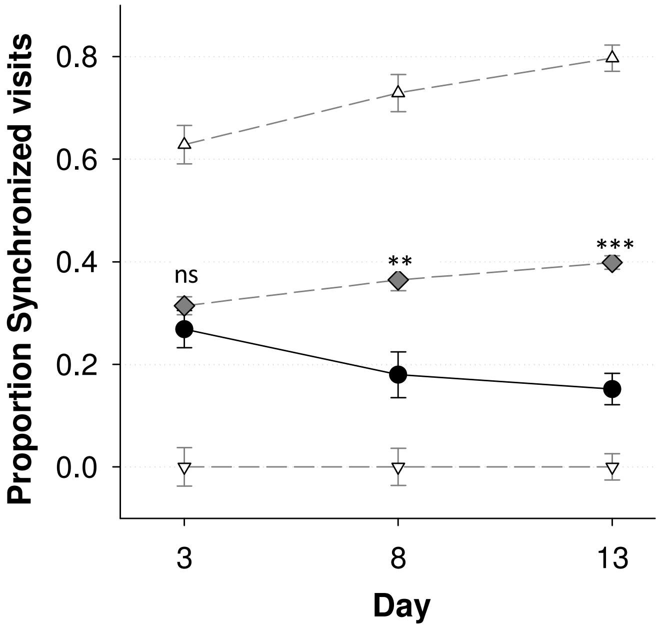

Supplementary Figure 1: **Temporal variation in population mean (± SE) nest visit synchronization as a proxy for parental cooperation.** The average between the theoretical maximal (white triangles pointing up) and minimal (white triangles pointing down) value was calculated for each observation and is considered the expected proportion of synchronized nest visits by chance (grey diamond symbols), given the observed parental nest visits. Observed synchronization scores (black dots, solid line) decreased with nestling age and were generally lower than the observed values. Daily Tukey corrected post-hoc differences between observed and expected samples are indicated as 'ns' (P>0.05), ** (0.01<P<0.001) and *** (P< 0.001).

Offspring growth, fledgling condition, fledgling body mass, NOx levels and nestling survival were not significantly affected by average synchronization levels (all $P \geq 0.16$) and neither by changes in synchronization levels from day 3 until day 13 (all $P \geq 0.25$). All other predictor variables had very similar effects as described for the analyses that included alternation (see main manuscript).

Conclusion

We found that the degree of synchronization strongly decreased with nestling age, which corresponds with the temporal pattern of female brooding. When females were on the nest and food was received by the male, females often directly transferred this received food to their offspring (A.I. personal observation). This frequently stimulated the males to jointly provide food directly to the offspring, explaining the highest synchronization levels during our early observations. The enhanced thermoregulation of the developing nestlings allowed females to allocate more time towards other behaviours, which may have reduced the trigger for males to jointly feed their nestlings. Synchronization in canaries can thus be summarized as a behavioural by-product of female brooding behaviour.

Similar as for the results on alternation, both overall and changing levels of synchronization have no effect on any of our offspring fitness estimates. The implications of these finding are discussed in the main article.

Supplementary material 2 – Behavioural repeatability

Statistical approach

The mixed models that explore temporal and sex-specific variation in the behavioural traits allowed to partition the between-individual from the within-individual variance through the random effect of BirdID[4]. This provides useful information about the temporal consistency of behavioural and cooperative parental strategies. We focused on the period of postnatal care and hence excluded the data collected during incubation. The repeatability of a given behavioural trait was then calculated as the between-individual variance upon the total variance, i.e. between- and within-individual variance[4]. The sim function (arm package)[5] was used to simulate values (10.000 iterations) of the posterior distribution for all model parameters and 95% credible intervals (CI).

Results

All six behavioural activities showed repeatable variation, with repeatability estimates being highest for foraging and resting, intermediate for offspring provisioning and brooding, and lowest for mate provisioning and singing behaviour (see Supplementary Table 1). These repeatability estimates were also calculated for each sex separately. Offspring provisioning was slightly less repeatable for males (R = 0.251, 95% CI [0.159-0.365], N = 31), compared to females (R = 0.306 [0.203-0.428], N = 31). This contrasts with foraging and resting behaviour, for which repeatability was higher in males, relative to females (foraging: males, R = 0.707 [0.554-0.820]; females, R = 0.484 [0.308-0.662]; resting: males, R = 0.788 [0.663-0.873]; females: R = 0.167 [0.076-0.316]; all N = 11). Repeatability for the estimates of cooperation were low; alternation (R = 0.148) and synchronization (R = 0.0134, see Supplementary Table 1).

Supplementary Table 1: Overview of the behavioural parameters. Mean (± SE) time per hour allocated to six different behavioural traits and mean (± SE) percentage of alternated and synchronized nest visits. Sample size (N) indicates the number of individuals/couples for which each parameter was quantified three times (day 3, 8 and 13). Between (V_{ind})- and within (V_0)-individual/couple variances (± 95% credible intervals) are derived from the LMMs, from which the adjusted repeatability values (± 95% credible intervals) were calculated. Song behaviour was square root transformed to meet the model assumptions.

Parameter	Mean ± SE	N	V_{ind} (between)	V_0 (within)	Repeatability
Offspring provisioning	1.64 ± 0.08	62	0.33 [0.25 - 0.44]	0.63 [0.53 - 0.77]	0.34 [0.26 - 0.43]
Mate provisioning	0.52 ± 0.05	31	0.01 [0.01 - 0.02]	0.18 [0.14 - 0.24]	0.07 [0.04 - 0.12]
Brooding	30.5 ± 1.70	31	73.02 [50.33 - 103.9]	124.58 [96.50 - 165.8]	0.37 [0.26 - 0.49]
Foraging	24.4 ± 1.40	22	50.74 [34.32 - 74.78]	36.40 [27.41 - 49.99]	0.58 [0.44 - 0.70]
Resting	18.4 ± 1.07	22	29.00 [19.71 - 42.34]	28.94 [21.76 - 39.51]	0.50 [0.36 - 0.63]
Singing	0.42 ± 0.11	11	0.07 [0.037 - 0.14]	0.42 [0.29 - 0.66]	0.14 [0.07 - 0.28]
Alternation (%)	59.2 ± 1.78	31	0.44 [0.28 - 0.69]	2.53 [1.94 - 3.37]	0.15 [0.09 - 0.24]
Synchronization (%)	20.8 ± 2.20	31	0.16 [0.10 - 0.26]	4.46 [3.43 - 5.96]	0.03 [0.02 - 0.06]

Conclusion

Our three repeated measurements span a large part of the post-hatching period of parental care. The observed repeatability was low ($R < 0.3$) for mate provisioning, song behaviour and both estimates for parental cooperation, moderate ($0.3 \leq R \leq 0.5$) for direct offspring provisioning, brooding and resting, and high ($R > 0.5$) for foraging behaviour. Thus the extent to which individuals are consistent in their behaviour, depends on the specific trait under consideration. Furthermore, the observed repeatability values in the current study are generally lower than earlier reports for similar behavioural traits in our population[6,7]. This might be explained mainly by the longer period between the repeated measurements in the current study[8]. Given these behavioural repeatability estimates, combined with the described temporal patterns in the main article, we strongly recommend to include temporal dynamics of behaviour in future studies, and to carefully consider the time windows between repeated measurements[8].

References

1. Mariette, M. M. & Griffith, S. C. The adaptive significance of provisioning and foraging coordination between breeding partners. *Am. Nat.* **185,** 270–280 (2015).

2. van Rooij, E. P. & Griffith, S. C. Synchronised provisioning at the nest: parental coordination over care in a socially monogamous species. *PeerJ* **1,** e232 (2013).

3. Bebbington, K. & Hatchwell, B. J. Coordinated parental provisioning is related to feeding rate and reproductive success in a songbird. *Behav. Ecol.* **27,** 652–659 (2016).

4. Dingemanse, N. J. & Dochtermann, N. A. Quantifying individual variation in behaviour: mixed-effect modelling approaches. *J. Anim. Ecol.* **82,** 39–54 (2013).

5. Gelman, A. *et al.* arm: data analysis using regression and multilevel/hierarchical models. R package version. 1–3 (2015).

6. Iserbyt, A., Eens, M., Baetens, W., Vermeulen, A. & Müller, W. Within- and between-individual (co)variance partitioning reveals limited pleiotropic effects of testosterone on immune function, sexual signaling and parental investment. *Behav. Ecol. Sociobiol.* **71,** 74 (2017).

7. Trösch, M., Müller, W., Eens, M. & Iserbyt, A. Genes, environments and their interaction: song and mate choice in canaries. *Anim. Behav.* **126,** 261–269 (2017).

8. Bell, A. M., Hankison, S. J. & Laskowski, K. L. The repeatability of behaviour: a meta-analysis. *Anim. Behav.* **77,** 771–783 (2009).
